# Formononetin ameliorates polycystic ovary syndrome through suppressing NLRP3 inflammasome

**DOI:** 10.1186/s10020-025-01092-x

**Published:** 2025-01-27

**Authors:** Zhuo Liu, Rui-Han Wang, Ke-Hua Wang

**Affiliations:** 1 Reproduction and Genetics Center, Affiliated Hospital of Shandong University of Traditional Chinese Medicine, 42 Wenhua West Road, Lixia District, Jinan, 250014 Shandong China; 2 The First Clinical College, Shandong University of Traditional Chinese Medicine, Jinan, 250355 Shandong China

**Keywords:** Formononetin, PCOS, NLRP3 inflammasome, inflammation

## Abstract

**Background:**

Polycystic ovary syndrome (PCOS) is a common gynecological disease accompanied by multiple clinical features, including anovulation, hyperandrogenism, and polycystic ovarian morphology, leading to infertility. Formononetin (FMN), which is a major bioactive isoflavone compound in *Astragalus membranaceus*, exerts anti-inflammatory effects. However, whether FMN is effective in the treatment of PCOS remains unknown. This study aims to explore the effects and the possible mechanisms of FMN in PCOS.

**Methods:**

Dehydroepiandrosterone (DHEA)-induced PCOS rats and dihydrotestosterone (DHT)-induced PCOS cell models were established. Fifty rats were randomly assigned into five groups of 10 rats each: Control, PCOS, PCOS + FMN (15 mg/kg), PCOS + FMN (30 mg/kg), and PCOS + FMN (60 mg/kg). Fasting blood glucose, insulin, luteinizing hormone, follicle-stimulating hormone, testosterone, and estradiol were detected in DHEA-induced PCOS rats. Ovarian histological changes and apoptosis were evaluated utilizing H&E and TUNEL staining. Subsequently, the effects of FMN on oxidative stress and inflammatory responses in the DHEA-induced PCOS rat model and DHT-induced PCOS cell model were explored. Besides, the function of FMN on cell viability and apoptosis in DHT-induced PCOS cell model were explored by using CCK-8 assay and flow cytometry. Protein expression was detected via western blot and immunofluorescence staining in the DHEA-induced PCOS rat model and DHT-induced PCOS cell model.

**Results:**

FMN alleviated PCOS symptoms and reduced inflammation, cell apoptosis, and oxidative stress in DHEA-induced PCOS rats and DHT-induced KGN cells. Additionally, FMN suppressed NLRP3 inflammasome activation in both models. In the DHT-induced PCOS cell model, nigericin (a activator of NLRP3) reversed the functions of FMN on inflammation, apoptosis, and oxidative stress.

**Conclusion:**

These findings demonstrated that FMN could alleviate PCOS by repressing inflammation, apoptosis, as well as oxidative stress in vivo and in vitro via inhibition of the NLRP3 inflammasome.

**Highlights:**

1. FMN improved PCOS symptoms.
2. FMN alleviated cell apoptosis, inflammation and oxidative stress in PCOS.
3. FMN inhibited the activation of NLRP3 inflammasome in PCOS.

## Introduction

Polycystic ovary syndrome (PCOS) is a widespread endocrine and metabolic disorder among reproductive-aged women, affecting 12 to 18% of this population depending on the diagnostic criteria and demographics studied (Kiani et al. 2022; Wolf et al. 2018; Alesi et al. 2022). Abnormal ovulation, hyperandrogenism, polycystic ovarian morphology, and insulin resistance (IR) are the hallmark features of PCOS (Meczekalski et al. 2023; Teede et al. 2018). PCOS represents a substantial health burden for women and poses a considerable economic challenge to communities (Teede et al. 2010). Besides, studies have confirmed increased symptoms of depression and anxiety in patients with PCOS (Dokras et al. 2012, 2016; Barry et al. 2011). Consequently, research on therapeutic approaches and mechanisms underlying PCOS has garnered considerable attention in recent years; however, effective intervention strategies remain limited.

**Fig. 1:**
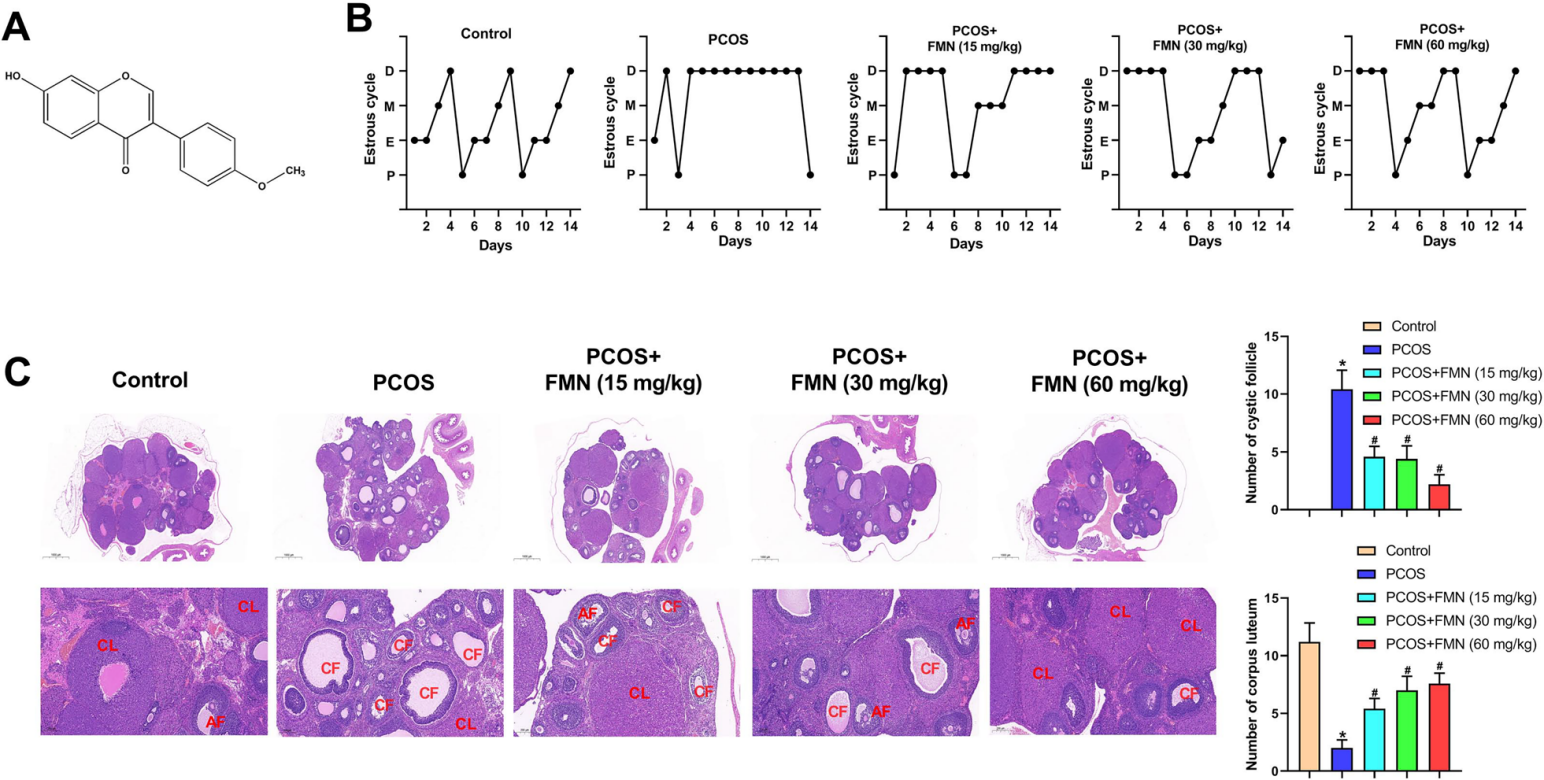
FMN improved the symptoms of PCOS in rats. **A** Chemical structure of FMN; (**B**) Representative pictures of estrous cycle in five different groups (P: proestrus; E: estrus; M: metestrus; D: diestrus); (**C**) H&E staining was employed for assessing ovarian histological changes in different groups (CF: cystic follicle; CL: corpus luteum; AF: antral follicle). ^*^P < 0.05, ^#^P < 0.05

In recent years, the use of natural products in clinical settings has gained tremendous popularity (Ong et al. 2019). Formononetin (FMN), one of the primary isoflavonoid constituents, is predominantly found in the Chinese herb *Astragalus membranaceus* (Zhang et al. 2018a), and its chemical structure is shown in Fig. 1A. FMN has demonstrated anti-inflammatory, anti-apoptotic, and antioxidative properties in various diseases, including cerebral ischemia–reperfusion injury (Yu et al. 2022), atherosclerosis (Ma et al. 2020), oxaliplatin-induced peripheral neuropathy (Fang et al. 2020), diabetic nephropathy (Huang et al. 2022), and inflammatory bowel disease (Wu et al. 2020), through the regulation of diverse pathways. Recently, FMN has been proved to affect women’s health. For example, Park et al. (Park et al. 2023) have reported that FMN inhibits progression of endometriosis. Tung et al. (Tung et al. 2015) have suggested that FMN could contribute to the improved rate of in vitro* fertilization*. Moreover, FMN inhibits proliferation and metastasis of ovarian cancer cells (Zhang et al. 2018b). However, the protective functions and mechanisms of FMN on PCOS are yet to be studied.

The current study aims to explore the protective effects and underlying mechanisms of FMN in a dehydroepiandrosterone (DHEA)-induced PCOS rat model and a dihydrotestosterone (DHT)-induced PCOS cell model. Our findings demonstrated that FMN could alleviate inflammation, apoptosis, and oxidative stress in PCOS models in vivo and in vitro through inhibiting the NLRP3 inflammasome, identifying that FMN holds promise as an effective therapeutic agent for PCOS.

## Methods

### Rat PCOS model

Female prepuberal Sprague–Dawley rats (three-week-old) were purchased from Pengyue (Jinan, China). All rats were maintained under controlled conditions at an ambient temperature of 22–24 °C with 65 ± 5% humidity and a 12 h light/dark cycle for 7 days to acclimate to the environment. The experimental protocol of our study was performed in accordance with the Guide for the Care and Use of Laboratory Animals and approved by Affiliated Hospital of Shandong University of Traditional Chinese Medicine (SDSZYY-SZYYCK-YXLLSP-099).

Fifty rats were randomly assigned into five groups of 10 rats each: Control, PCOS, PCOS + FMN (15 mg/kg), PCOS + FMN (30 mg/kg), and PCOS + FMN (60 mg/kg). Rats subcutaneously injected with DHEA (60 mg/kg/day) for consecutive 21 days were assigned to the PCOS group, while those administered sesame oil were designated as the control group. After 21 days of DHEA treatment, FMN (15, 30 and 60 mg/kg) (Liu et al. 2021a) was administrated into the PCOS rats by oral gavage once daily for another 21 days. The control group and the PCOS group were given an equal volume of normal saline for 21 days. Finally, all rats were sacrificed, and ovarian tissue samples were collected. DHEA was procured from Sigma-Aldrich (MO, USA). FMN was supplied by Yuanye (shanghai, China).

### Estrous cycle detection

The oestrous cycle is constituted by proestrus (P), estrus (E), metestrus (M), and dioestrus (D). Vaginal smear analysis was performed daily between 9:00 to 10:00 A.M. from day 8 after FMN treatment until the end of the experiments. A sterile cotton swab dipped in normal saline gently scraped the cells on the vagina wall of the rats and evenly smeared them on the slide in a clockwise direction. The cycle stages were determined by visual inspection of cells under a microscope (NIKON, Japan).

### Detection of hormone levels

The levels of luteinizing hormone (LH), follicle-stimulating hormone (FSH), testosterone, and estradiol (E2) in serum were analyzed utilizing their responding ELISA kits based on the steps of instructions (Rat LH ELISA kit, SEKR-0091, Solarbio; Rat FSH ELISA kit, SEKR-0090, Solarbio; Testosterone ELISA kit, SEKSM-0003, Solarbio; Rat E2 ELISA kit, SEKR-0107, Solarbio).

### Insulin resistance assay

Fasting serum insulin (FINS) levels were determined using an insulin ELISA kit (SEKR-0033, Solarbio). Moreover, a blood glucose content assay kit (BC2495, Solarbio) was applied for measuring fasting blood glucose (FBG) level. Subsequently, the homeostasis model assessment of insulin resistance (HOMA-IR) was calculated using the formula: HOMA-IR = (FBG × FINS)/22.5.

### Histological analysis

Ovarian tissues were fixed in formalin for 24 h, and subsequently embedded by paraffin. Serial paraffin Sects. (4 μm thick) were stained according to the methods of hematoxylin–eosin staining.

For TUNEL staining, 4 μm-thick section was deparaffinized and dehydrated with gradient ethanol, followed by incubation using proteinase K for 20 min. Next, the section was incubated with TUNEL reaction solution at 37 °C for 1 h in darkness. Visualization was performed using 3,3′-diaminobenzidine (DAB, Sigma, USA), and TUNEL-positive cells were imaged under a microscope (NIKON, Japan).

### Detection of GSH, SOD and CAT contents

Supernatants from cell culture media and ovarian tissues were collected, and protein concentrations were detected utilizing BCA kit. Glutathione (GSH) content was determined applying a Reduced GSH Content Assay Kit (Solarbio, Beijing, China). Superoxide Dismutase (SOD) and Catalase (CAT) Activity Assay Kits (Solarbio) were utilized for evaluating the activities of SOD and CAT.

### RNA extraction and real-time PCR

Total RNA from KGN cells and ovarian tissues was extracted employing TRIzol reagent (Invitrogen, USA). One microgram of RNA was converted to cDNA utilizing a Revert Aid First Strand cDNA Synthesis kit (Thermo Scientific, USA). Then, the expression of indicated genes was quantified by real-time PCR with the UltraSYBR One Step RT-qPCR kit (Cwbio, Jiangsu, China), using β-actin as the endogenous control. The primer sequences were as follows: TNF-α-rat F, GATCGGTCCCAACAAGGAGG, R,

CTTGGTGGTTTGCTACGACG; IL-1β-rat F,

ATAGCAGCTTTCGACAGTGAGG, R, TCTGGACAGCCCAAGTCAAG; IL-6-rat F, AGAGACTTCCAGCCAGTTGC, R, TGCCATTGCACAACTCTTTTC; β-actin-rat F, GCCTTCCTTCCTGGGTATGG, R, AATGCCTGGGTACATGGTGG; TNF-α-human F, GCCCATGTTGTAGCAAACCC, R, GGAGGTTGACCTTGGTCTGG; IL-1β-human F, GCCCTAAACAGATGAAGTGCT, R, GGTGGTCGGAGATTCGTAGC; IL-6-human F, ATGAACTCCTTCTCCACAAGCG, R, TGTTACTCTTGTTACATGTCTCCTT; β-actin-human F, GATTCCTATGTGGGCGACGA, R, AGGTCTCAAACATGATCTGGGT.

### Western blotting

Ovarian tissues and KGN cells were lysed using RIPA buffer (Santa Cruz Biotechnology, USA). The protein (25 μg) was denatured and resolved by 10% SDS-PAGE, transferred onto a nitrocellulose membrane, and incubated with primary antibodies including Bcl-2 (1:500, Cat.26593-1-AP, Proteintech, USA), Bax (1:1000, Cat.50599-2-Ig, Proteintech, USA), cleaved caspase 3 (1:500, Cat.#9661, Cell Signaling, USA), NLRP3 (1:500, Cat.ab263899, Abcam, UK), ASC (1:500, Cat.ab309497, Abcam, UK), caspase 1 (1:500, Cat.#83383, Cell Signaling, USA), GAPDH (1:5000, Cat.10494-1-AP, Proteintech, USA) overnight at 4 °C. After washing with PBST three times, the nitrocellulose membrane was incubated with HRP-conjugated anti-rabbit IgG (1:5000,Cat.ab270144, Abcam, UK) at room temperature for 120 min. A t last, protein bands were visualized by the ECL Western Blotting Substrate (Solarbio, Beijing, China).

### Cell culture and treatment

Human granulosa cells (KGN) were provided by Procell (Wuhan, China). The cells were grown in DMEM/F12 medium (HuanKai Biology, Guangdong, China) in the presence of 10% fetal bovine serum (Thermo Fisher Scientific, USA) and 1% penicillin/streptomycin (Sigma, USA) in a humidified incubator with 5% CO_2_ at 37 °C. The KGN cells were subsequently treated with DHT (500 nM, Selleck, USA) for 24 h as a PCOS cell model, as described previously (Ji et al. 2022; Dilaver et al. 2019; Zhou et al. 2020). FMN (12.5, 25, and 50 μmol/L) (Liu et al. 2021a) or Nigericin (a activator of NLRP3, MCE, USA) was administered for 24 h following DHT treatment. The control group and DHT group were given an equal volume of DMSO for 24 h.

### Evaluation of cell viability

KGN cell viability was observed utilizing CCK-8 (Beyotime, Shanghai, China). After different treatments, KGN cells were planted into 96-well plates and cultivated for 24 h. Subsequently, cells were incubated with CCK-8 solution (10 µL/well) at room temperature for 1.5 h. Finally, the absorbance at 450 nm was detected and recorded employing a microplate reader.

### Cell apoptosis assay

KGN cells were suspended in 100 μL binding buffer. Next, Annexin V-FITC (5 μL, BD Biosciences, USA) and propidium iodide (10 μL, Beyotime, Shanghai, China) were added to cell suspension and the mixture was incubated in darkness for 15 min. Finally, flow cytometry was employed for determining apoptotic rate.

### Immunofluorescent staining

Cells were fixed in 70% ethanol for 10 min at room temperature permeabilized with 0.1% Triton-100 in PBS for 20 min. After blocking with 5% bovine serum albumin in PBS, KGN cells were incubated with NLRP3 antibody (1:50, cat.27458-1-AP, Proteintech, USA) for 2 h at room temperature with gentle shaking. Subsequent secondary antibody was applied on the slide for 30 min. Followed by washed in PBS, KGN cells were stained using DAPI in darkness for 5 min. Finally, Slides were examined under a fluorescence microscope (NIKON, Japan).

### Bioinformatics analysis

Molecular docking is regarded as a key technique for structure-based research and drug development. FMN molecular structure was downloaded from the PubChem database for the subsequent MOE molecular docking. Moreover, the structure of NLRP3 protein was obtained from the RCSB PDB database and downloaded in PDB format. Next, MOE molecular docking software was used for molecular docking of FMN with NLRP3. In addition, KEGG enrichment analysis was used to explore the enriched pathways of FMN.

### Statistical analysis

Data are presented as the mean ± SEM of biological replicates. Statistical analyses were performed using GraphPad Prism 8.0 software. Statistical significance was tested using one-way ANOVA with Tukey’s post hoc test and P values of less than 0.05 were considered significant.

## Results

### FMN improved the symptoms of PCOS rats

**Fig. 2:**
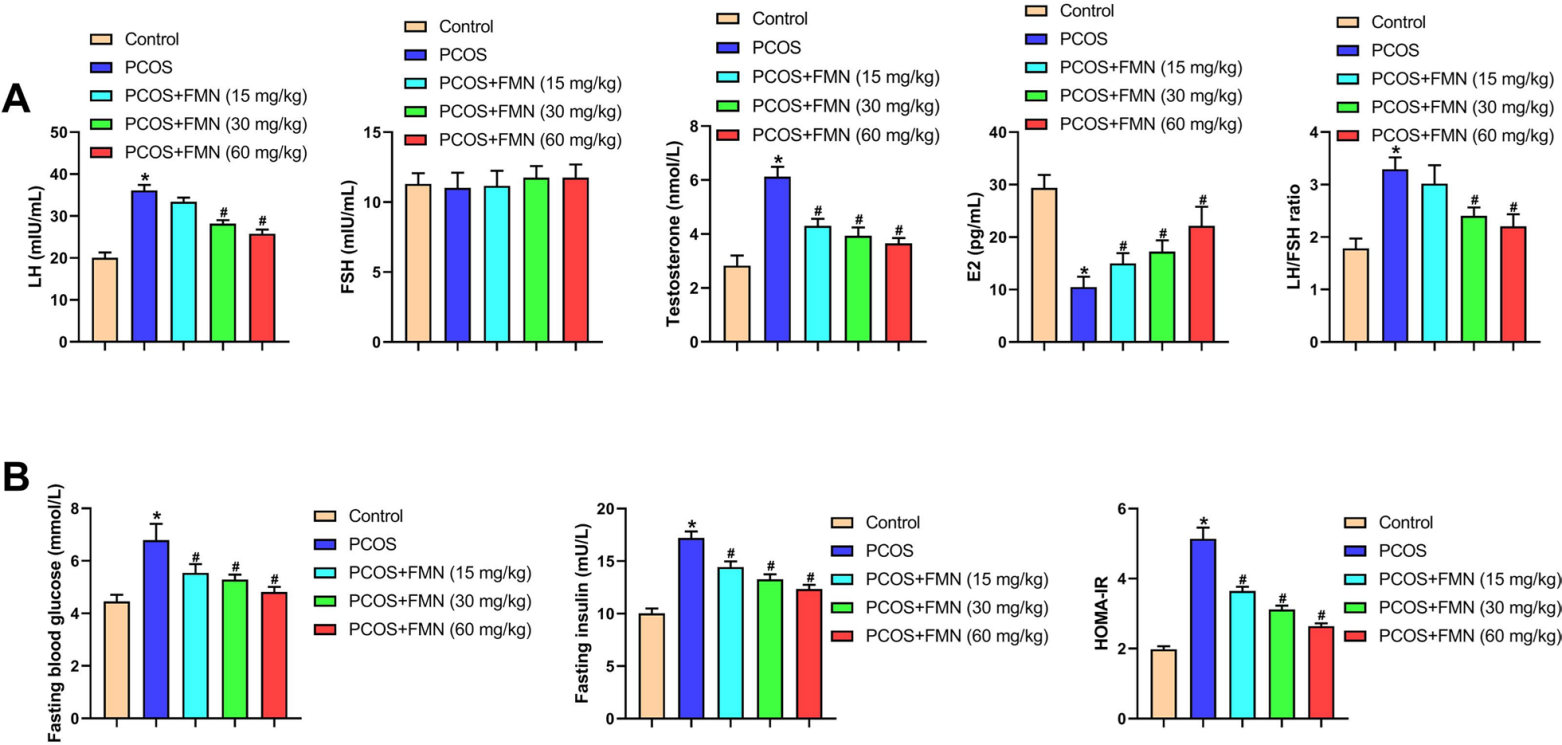
FMN regulated the levels of sexual hormones and inhibited IR in PCOS rats. **A** LH, FSH, Testosterone, E2, and the LH/FSH ratio in different groups; (**B**) Blood glucose indices (fasting blood glucose, fasting insulin and HOMA-IR) in different groups. ^*^P < 0.05, ^#^P < 0.05.

The chemical structure of FMN is shown in Fig. 1A. The estrus cycle is an important indicator of healthy ovary. Hence, we detected the estrus cycle utilizing vaginal smear. PCOS group rats lost the regular estrous cycle (Fig. 1B). After FMN treatment, the irregular estrous cycle was correct (Fig. 1B). HE staining showed the corpus luteum reduction, and cystic follicle increase in the PCOS group compared with the control group, and these phenotypes were improved after FMN treatment (Fig. 1C). Next, we further detected four sex steroid hormones levels (LH, FSH, testosterone and E2), and found that FMN administration markedly reversed the increased levels of LH, testosterone, and the LH/FSH ratio in PCOS rats, as well as the decreased level of E2 (Fig. 2A). However, FSH levels did not differ significantly among the groups (Fig. 2A). Then, the glucose metabolism was measured to confirm the roles of FMN on insulin resistance, a key feature of PCOS (Corbould et al. 2005). When compared with Control group, the fasting blood glucose, fasting insulin, and HOMA-IR were elevated in the PCOS group (Fig. 2B). FMN treatment significantly reduced fasting blood glucose, fasting insulin, and HOMA-IR levels (Fig. 2B). Collectively, these findings indicated that FMN improved PCOS symptoms in rats.

### FMN alleviated inflammation, cell apoptosis, and oxidative stress in DHEA-induced PCOS rats

**Fig. 3:**
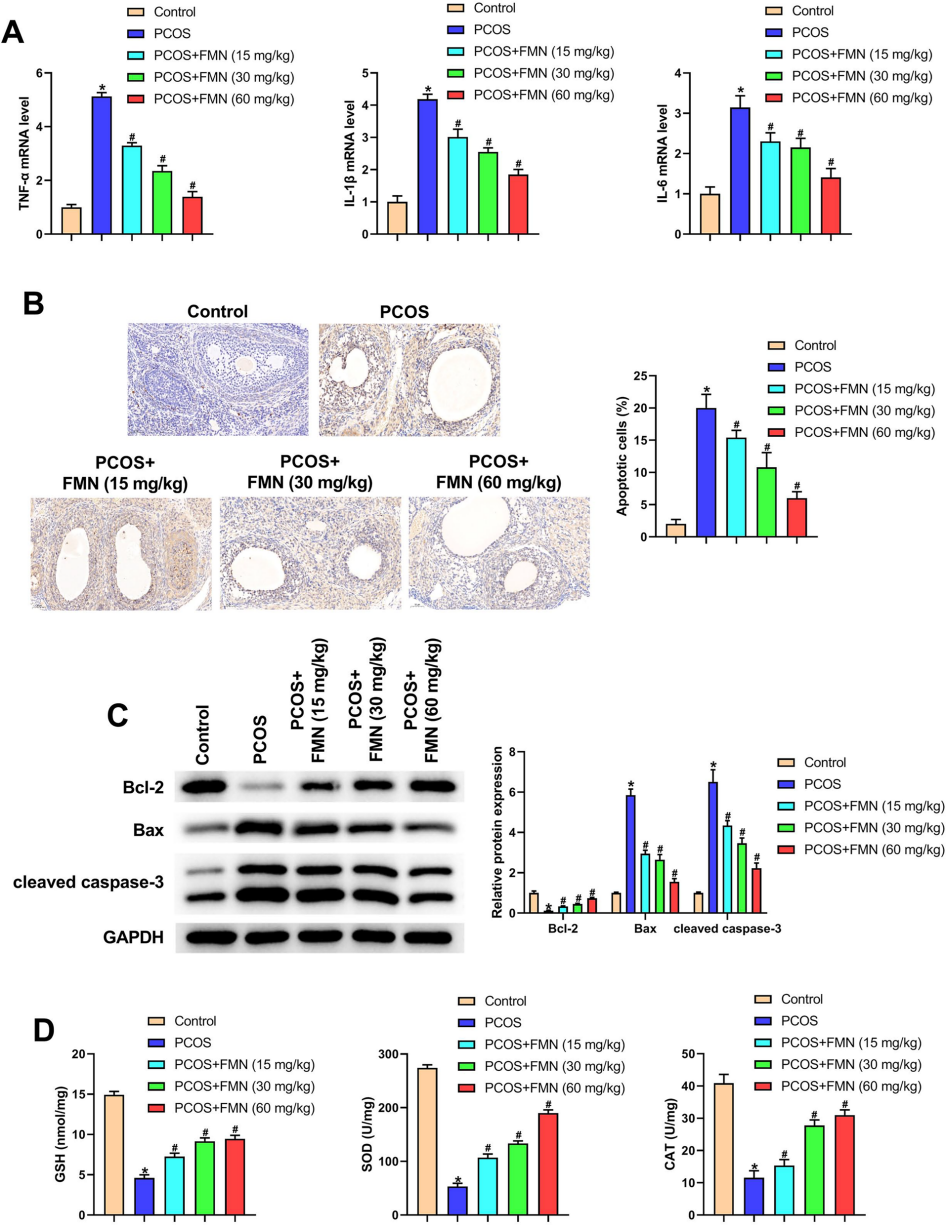
FMN alleviated inflammation, cell apoptosis, and oxidative stress in DHEA-induced PCOS rats. **A** TNF-α, IL-1β and IL-6 mRNA level in ovarian tissues was detected utilizing qRT-PCR in different groups; (**B**) TUNEL was utilized for assessing cell apoptosis in different groups of ovarian tissues; (**C**) Western blot was employed for measuring the apoptosis-related proteins in different groups of ovarian tissues; (**D**) GSH, SOD and CAT in different groups of ovarian tissues. ^*^P < 0.05, ^#^P < 0.05

As seen in Fig. 3A, the upregulated levels of TNF-α, IL-1β and IL-6 in PCOS rats were reversed after FMN administration. Besides, TUNEL staining results showed that ovarian cell apoptosis was significantly elevated in DHEA-induced PCOS rats, while the increased apoptosis was notably abolished by FMN administration (Fig. 3B). Analysis of apoptosis-related proteins revealed that FMN administration restored the reduced Bcl-2 levels and reversed the elevated Bax and cleaved caspase-3 levels in PCOS rats (Fig. 3C). In addition, the data of Fig. 2D showed that GSH content and the activities of SOD and CAT were decreased in PCOS group rats compared with Control group rats. Interestingly, their decreased contents and activities were markedly reversed after the administration of FMN (Fig. 3D). The experimental results implied that FMN could alleviate inflammation, cell apoptosis and oxidative stress in DHEA-induced PCOS rats.

### FMN inhibited the activation of the NLRP3 inflammasome in DHEA-induced PCOS rats

**Fig. 4:**
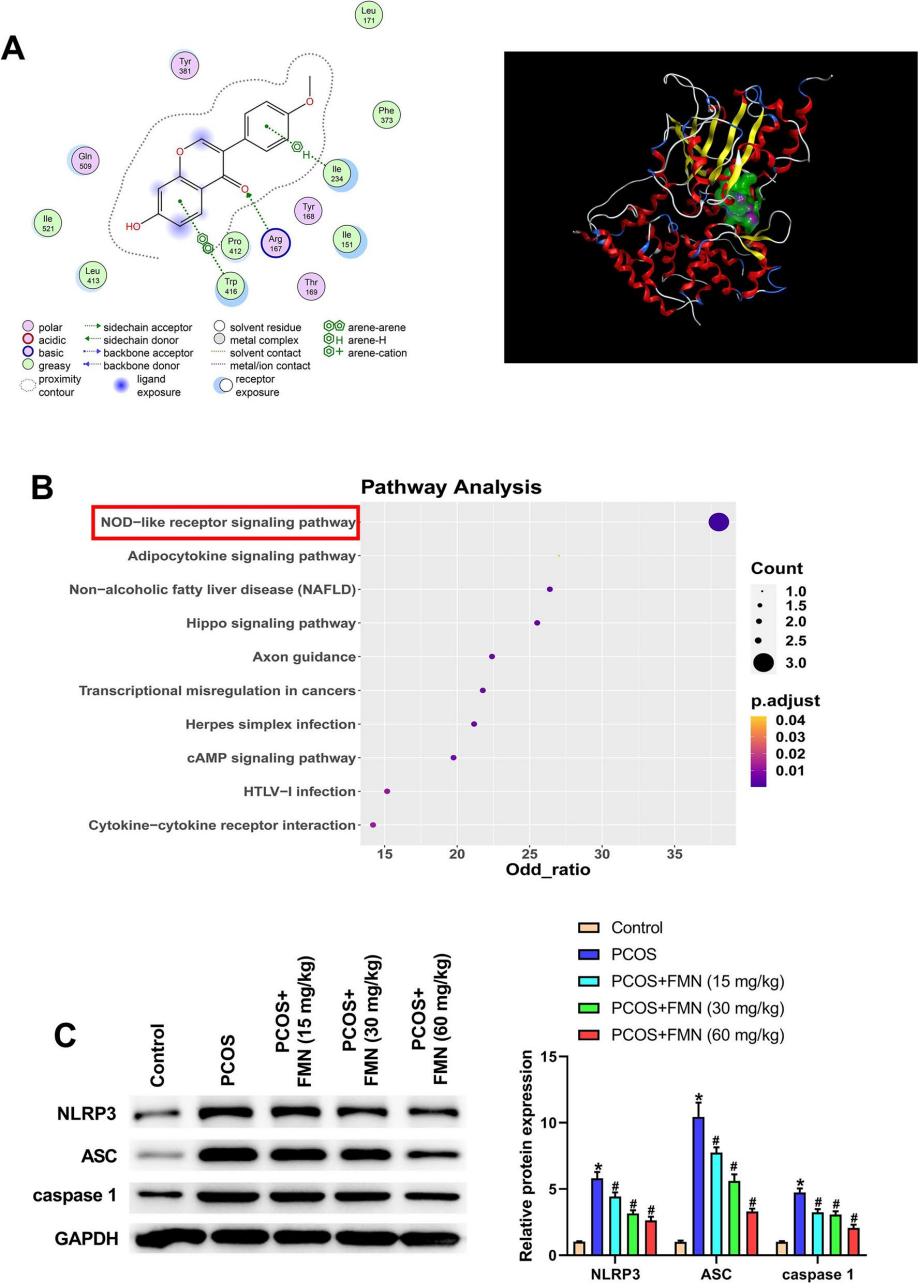
FMN inhibited the activation of the NLRP3 inflammasome in DHEA-induced PCOS rats. (**A**) Molecular docking mode of FMN and NLRP3 (affinity = -6.6161 kcal/mol); (**B**) KEGG enrichment analysis confirmed the association between the NOD-like receptor signaling pathway and FMN; (**C**) Western blot was employed for measuring NLRP3, ASC and caspase-1 levels in different groups. ^*^P < 0.05, ^#^P < 0.05

The result of molecular docking confirmed that FMN had a good binding affinity with NLRP3 (affinity = − 6.6161 kcal/mol), as shown in Fig. 4A. KEGG enrichment analysis further confirmed that the relationship between the NOD-like receptor signaling pathway and FMN (Fig. 4B). Therefore, we investigated the function of FMN on NLRP3 inflammasome in DHEA-induced PCOS rats, and observed that the upregulated expressions of NLRP3, ASC and caspase-1 caused by DHEA were notably abolished after the administration of FMN (Fig. 4C). To sum up, FMN could inhibit the activation of NLRP3 inflammasome in DHEA-induced PCOS rats.

### FMN alleviated inflammation, apoptosis, and oxidative stress in DHT-induced PCOS cell model

**Fig. 5:**
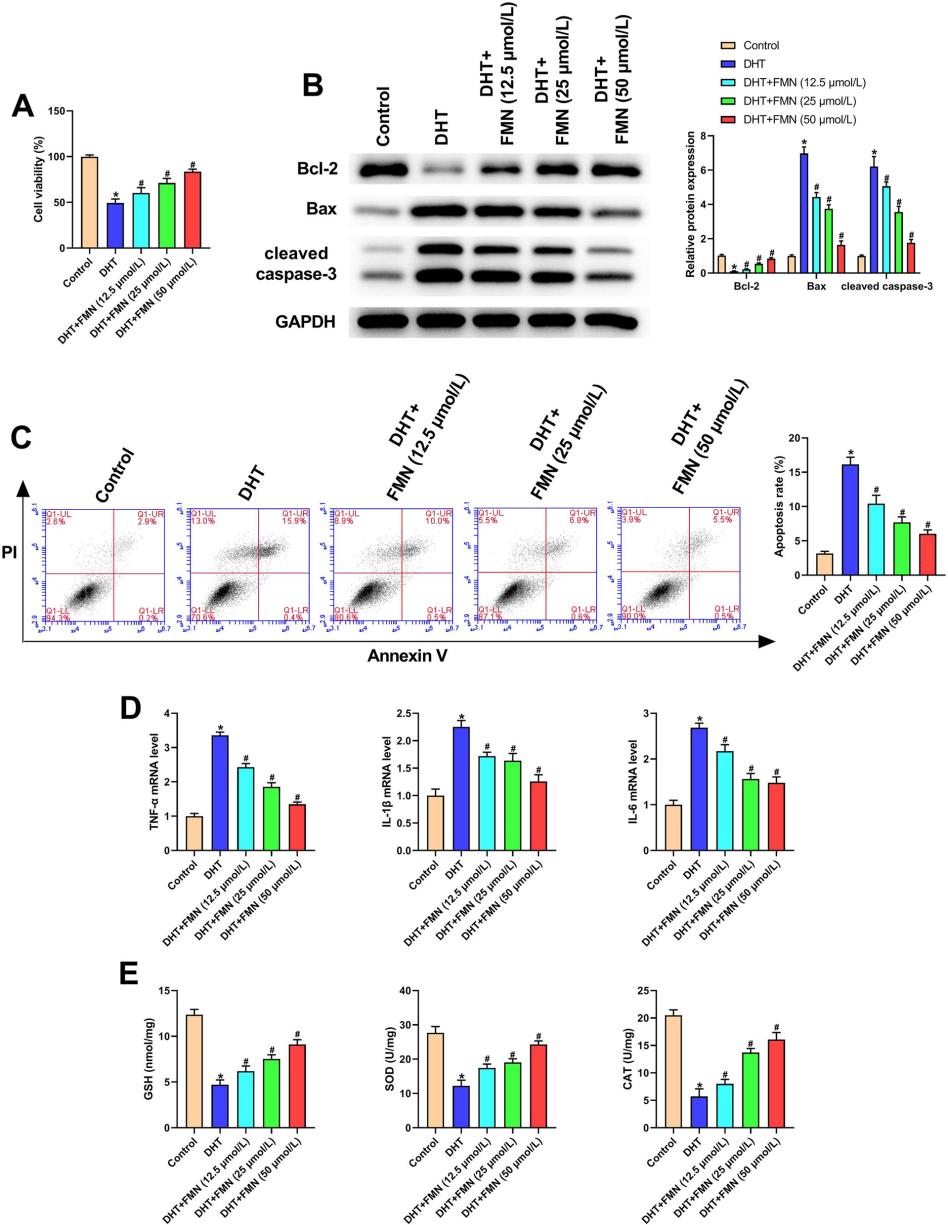
FMN alleviated cell apoptosis, inflammation, as well as oxidative stress in the DHT-induced PCOS cell model. **A** Cell viability was explored via employing CCK-8 assay in different groups; (**B**) Western blot was employed for measuring the apoptosis-related proteins in different groups; (**C**) Flow cytometry was utilized for assessing cell apoptosis ability in different groups. **D** TNF-α, IL-1β and IL-6 mRNA level in different groups was detected utilizing qRT-PCR; (**E**) GSH, SOD and CAT levels in different groups.^*^P < 0.05, ^#^P < 0.05

Figure 5A results manifested that the reduced cell viability caused by DHT was improved after FMN treatment. The expression of apoptosis-related proteins revealed downregulated Bcl-2 and upregulated Bax and cleaved caspase-3 in the DHT group, which were reversed by FMN treatment (Fig. 5B). Additionally, Fig. 5C data further verified the elevated apoptosis of KGN cells induced by DHT could be suppressed after FMN administration. Besides, FMN administration significantly reversed the upregulated levels of inflammatory factors in DHT-treated KGN cells (Fig. 5D). In addition, we also demonstrated that FMN treatment markedly attenuated the reduced GSH content, and SOD and CAT activity in DHT-induced PCOS cell model (Fig. 5E). These above observations indicated that FMN could alleviate inflammation, apoptosis, and oxidative stress in DHT-treated KGN cells.

### FMN inhibited the activation of the NLRP3 inflammasome in DHT-induced KGN cells

**Fig. 6:**
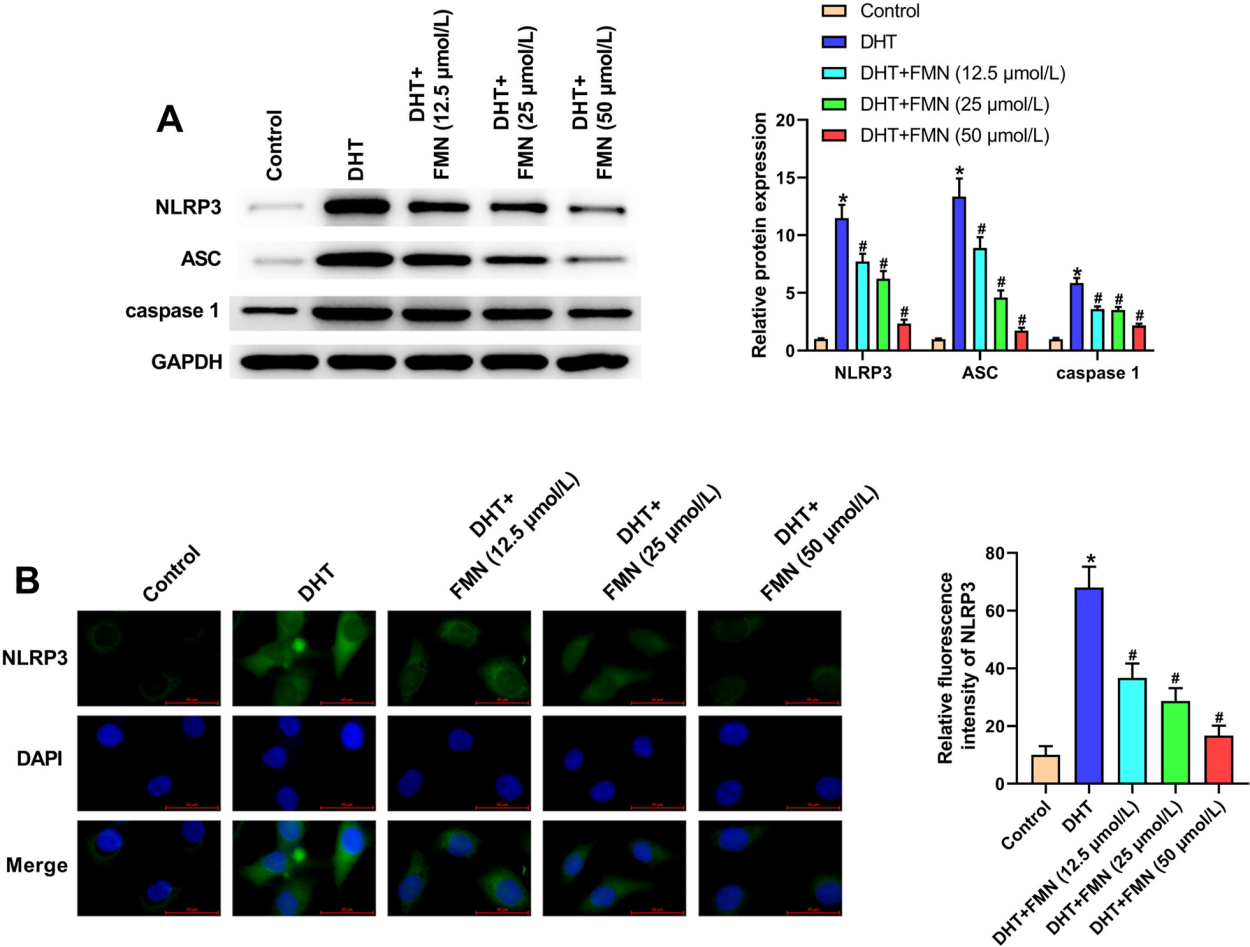
FMN inhibited the activation of the NLRP3 inflammasome in DHT-induced KGN cells. **A** Western blot was employed for measuring NLRP3, ASC and caspase-1 levels in different groups of KGN cells; (**B**) Immunofluorescence staining was utilized for detecting NLRP3 in different groups of KGN cells. ^*^P < 0.05, ^#^P < 0.05

The results of Fig. 6A showed that the expression of NLRP3, ASC and caspase-1 was significantly upregulated in the DHT group compared with the control group. When compared with DHT group, NLRP3, ASC and caspase-1 expression was markedly decreased after FMN administration (Fig. 6A). At the same time, immunofluorescent staining confirmed that FMN administration significantly suppressed the DHT-induced upregulation of NLRP3 expression in KGN cells (Fig. 6B). Collectively, FMN could inhibit the activation of the NLRP3 inflammasome in DHT-induced PCOS cell models.

### Nigericin abolished the effects of FMN on inflammation, apoptosis, and oxidative stress in DHT-induced KGN cells

**Fig. 7:**
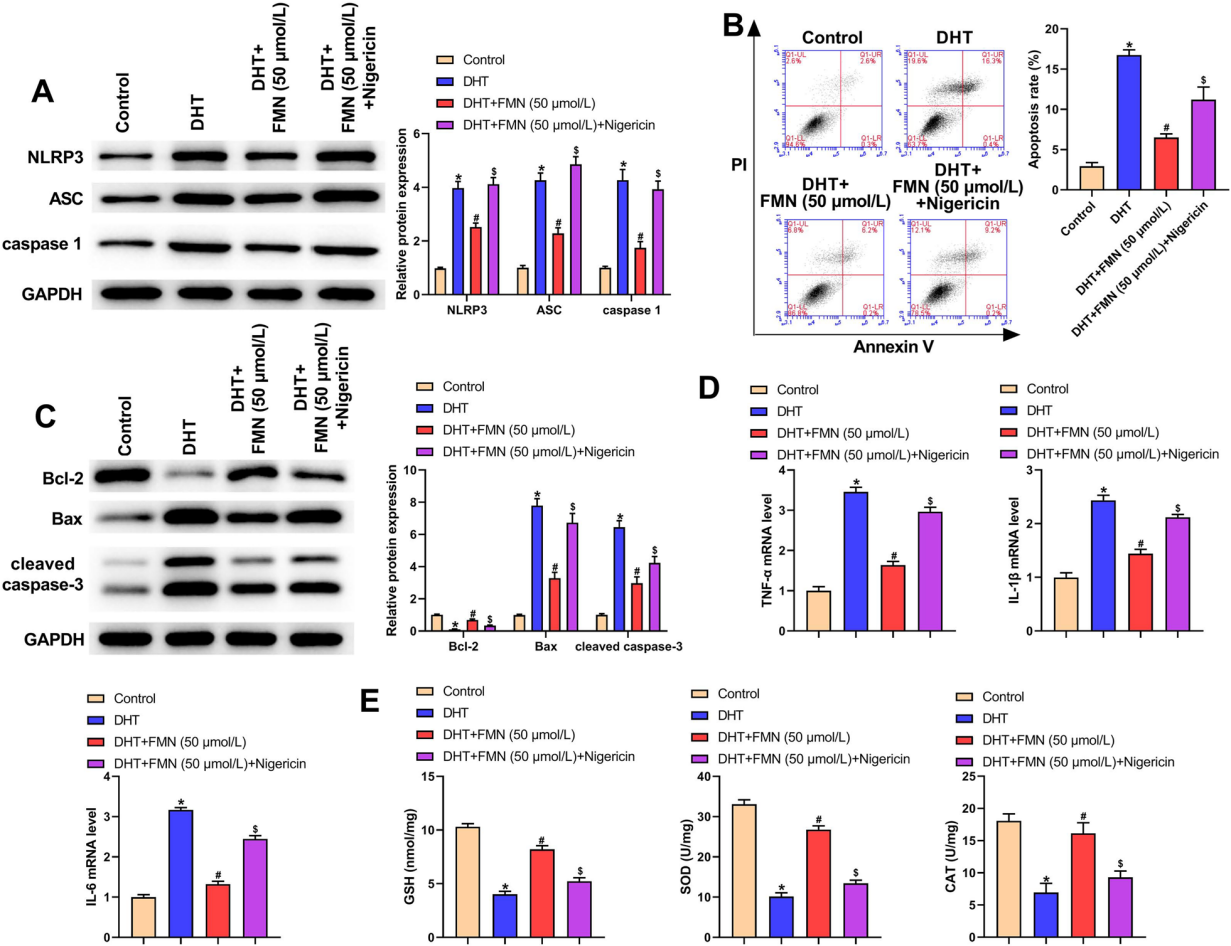
Nigericin abolished FMN functions on inflammation, apoptosis, and oxidative stress in the DHT-caused PCOS cell model. **A** Western blot was employed for measuring NLRP3, ASC and caspase-1 levels in different groups of KGN cells; (**B**) Flow cytometry was utilized for assessing cell apoptosis in different groups of KGN cells; (**C**) Western blot was employed for measuring the apoptosis-related proteins in different groups of KGN cells; (**D**) TNF-α, IL-1β and IL-6 mRNA level in different groups was detected utilizing qRT-PCR; (**E**) GSH, SOD and CAT levels in different groups of KGN cells. ^*^P < 0.05, ^#^P < 0.05, ^$^P < 0.05

As seen in Fig. 7A, the expression of NLRP3, ASC, and caspase-1 in the DHT + FMN (50 μmol/L) + Nigericin group was increased relative to the DHT + FMN (50 μmol/L) group. Flow cytometry demonstrated increased apoptosis in the DHT + FMN (50 μmol/L) + Nigericin group compared with the DHT + FMN (50 μmol/L) group (Fig. 7B). Moreover, western blot analysis revealed that Bcl-2 expression was significantly reduced, while Bax and cleaved caspase-3 levels were elevated in the DHT + FMN (50 μmol/L) + Nigericin group (Fig. 7C). Besides, TNF-α, IL-1β and IL-6 level was higher in the DHT + FMN (50 μmol/L) + Nigericin group than those in the DHT + FMN (50 μmol/L) group (Fig. 7D). When compared with the DHT + FMN (50 μmol/L) group, GSH content as well as the activities of SOD and CAT were markedly decreased in the DHT + FMN (50 μmol/L) + Nigericin group (Fig. 7E). Overall, these results indicated that Nigericin could abolish the beneficial effects of FMN on inflammation, apoptosis, and oxidative stress in a DHT-induced PCOS cell model.

## Discussion

PCOS seriously impacts the physical and mental health of patients. At present, the treatment of PCOS is a primary focus of research on reproductive diseases. In our study, we demonstrated that FMN could alleviate inflammation, apoptosis, and oxidative stress in PCOS both in vivo and/or in vitro through inhibiting the NLRP3 inflammasome.

Recently, FMN has been proved to affect women’s health. For example, Park et al. (Park et al. 2023) have reported that FMN inhibits progression of endometriosis. Tung et al. (Tung et al. 2015) have suggested that FMN could contribute to the improved rate of in vitro fertilization. Moreover, FMN inhibits proliferation and metastasis of ovarian cancer cells (Zhang et al. 2018b). This study is the first to clarify the therapeutic role of FMN in rats. The results confirmed that FMN treatment alleviated pathological changes and improved hormonal imbalances in a dose-dependent manner. Potential mechanisms underlying PCOS include intrinsic insulin resistance (Moghetti 2016). Moreover, Escobar et al. (Jatzko and Ott 2011) have proved that inflammation is a critical contributor to insulin resistance in women with PCOS. Growing evidence has manifested that chronic inflammation and oxidative stress contribute insulin resistance in PCOS (Murri et al. 2013; Cui et al. 2022). PCOS is characterized by an increased white blood cell count and elevated levels of IL-1β, IL-6, and TNF-α (Armanini et al. 2022). Emerging evidence has reported that oxidative stress contributes greatly to the progression of PCOS pathophysiology (Lu et al. 2018). Additionally, the levels of MDA, CAT, SOD and GPx are verified to be key factors in PCOS therapy (Cui et al. 2022). In this study, we confirmed that FMN alleviated inflammation and oxidative stress in DHEA-induced PCOS rats and DHT-induced PCOS cells via repressing inflammatory factors level and increasing GSH, SOD and CAT contents in a dose-dependent manner. Furthermore, the high concentration of insulin, which may simulate insulin resistance, can lead to the elevated apoptosis of ovarian granulosa cells in PCOS (Ni et al. 2015). Our findings suggested that FMN could alleviate cell apoptosis in DHEA-induced PCOS rats and DHT-induced PCOS cells in a dose-dependent manner.

Inflammasomes, a term first proposed by Tschopp in 2002 (Martinon et al. 2002), have been considered to participate the pathogenesis of a variety of chronic inflammatory responses and metabolic disorders. NLRP3 is an important cytoplasmic pattern recognition receptor that recruits apoptosis-associated speck-like protein (ASC) and caspase-1 to form inflammasomes that can induce inflammation and inflammatory cell death (Man and Kanneganti 2016; Zhao and Zhao 2020). Once assembled and activated, the NLRP3 inflammasome causes pro-caspase-1 self-cleavage and activation, which leads to the maturation of IL-1β and IL-18 (Huang et al. 2021). In recent years, more and more researches have verified the relationship between the NLRP3 inflammasome and PCOS. For example, Rubus chingii Hu could relieve PCOS by enhancing insulin sensitivity through suppression of the TXNIP/NLRP3 inflammasome pathway (Li et al. 2023). Zhou et al. (Zhou et al. 2023) have proved that metformin inhibits pyroptosis and inflammation via modulating the ROS/NLRP3 pathway in ovarian granulosa cells. Moreover, miR-1224-5p is reported to attenuate PCOS through inhibiting NLRP3 inflammasome activation via targeting forkhead box O1 (Li et al. 2021). Interestingly, FMN is proved to have anti-apoptotic and anti-inflammatory properties through suppression of the NLRP3 inflammasome in mice with autoimmune hepatitis (Liu et al. 2021b). Wu et al. (Wu et al. 2018) have demonstrated that FMN treatment can ameliorate dextran sulfate sodium-induced acute colitis through repressing the NLRP3 inflammasome. Through bioinformatic analysis, we concluded that the role of FMN in PCOS may be related to the NLRP3 inflammasome. Thus, we investigated whether FMN alleviates PCOS through the NLRP3 inflammasome. Using western blot and immunofluorescence staining, we observed that FMN treatment reversed the upregulated expression of NLRP3, ASC and caspase-1 induced by DHEA and DHT in rats and cells, indicating that FMN administration could inhibit NLRP3 inflammasome activation in PCOS rat and cell models. Subsequently, cell rescue experiments indicated that nigericin could abolish the functions of FMN on inflammation, apoptosis, and oxidative stress in a DHT-caused PCOS cell model.

Taken together, this study provides in vivo and in vitro evidence that FMN could alleviate the development of PCOS by repressing inflammation, apoptosis, and oxidative stress through inhibition of the NLRP3 inflammasome. Our findings suggested that FMN may be a promising candidate for PCOS treatment.

## Data Availability

The datasets used and analyzed during the current study are available from the corresponding author on reasonable request.
